# Comparison of Mesh Fixation Techniques in Elective Laparoscopic Repair of Incisional Hernia-ReliaTack™ v ProTack™ (TACKoMesh) - A double-blind randomised controlled trial

**DOI:** 10.1186/s12893-018-0378-3

**Published:** 2018-07-11

**Authors:** Aali J. Sheen, J. James Pilkington, Minas Baltatzis, Ahmed Tyurkylmaz, Panagiotis Stathakis, Saurabh Jamdar, Ajith K. Siriwardena

**Affiliations:** 1 Department of Surgery, Manchester University Foundation NHS trust, M13 9WL, Manchester, UK; 2 Department of Biomedicine, Manchester Metropolitan University, Manchester, UK; 3 Department of Hepatobiliary and Hernia Surgery, Manchester University Foundation NHS Trust, Oxford Road, Manchester, M13 9WL UK; 4 Fortius Clinc, 17 Fitzhardinge Street, London, W1H 6EQ UK

**Keywords:** Laparoscopic incisional hernia repair, Fascial closure, Closure of the fascial defect, Intraperitoneal onlay mesh, SymbotexTM composite mesh, Absorbable tacks, ReliaTackTM

## Abstract

**Background:**

Minimally invasive incisional hernia repair has been established as a safe and efficient surgical option in most centres worldwide. Laparoscopic technique includes the placement of an intraperitoneal onlay mesh with fixation achieved using spiral tacks or sutures. An additional step is the closure of the fascial defect depending upon its size. Key outcomes in the evaluation of ventral abdominal hernia surgery include postoperative pain, the presence of infection, seroma formation and hernia recurrence. TACKoMESH is a randomised controlled trial that will provide important information on the laparoscopic repair of an incisional hernia; 1) with fascial closure, 2) with an IPOM mesh and 3) comparing the use of an articulating mesh-fixation device that deploys absorbable tacks with a straight-arm mesh-fixation device that deploys non-absorbable tacks.

**Methods:**

A prospective, single-centre, double-blinded randomised trial, TACKoMESH, will establish whether the use of absorbable compared to non-absorbable tacks in adult patients undergoing elective incisional hernia repair produces a lower rate of pain both immediately and long-term. Eligible and consenting patients will be randomized to surgery with one of two tack-fixation devices and followed up for a minimum one year. Secondary outcomes to be explored include wound infection, seroma formation, hernia recurrence, length of postoperative hospital stay, reoperation rate, operation time, health related quality of life and time to return to normal daily activity.

**Discussion:**

With ongoing debate around the best management of incisional hernia, continued trials that will add substance are both necessary and important. Laparoscopic techniques have become established in reducing hospital stay and rates of infection and report improvement in some patient centered outcomes whilst achieving similarly low rates of recurrence as open surgical techniques. The laparoscopic method with tack fixation has developed a reputation for its tendency to cause post-operative pain. Novel additions to technique, such as intraoperative-sutured closure of a fascial defect, and developments in surgical technology, such as the evolution of composite mesh design and mesh-fixation devices, have brought about new considerations for patient and surgeon. This study will evaluate the efficacy of several new technical considerations in the setting of elective laparoscopic incisional hernia repair.

**Trial registration:**

Name of registry - ClinicalTrials.gov Registration number: NCT03434301. Retrospectively registered on 15th February 2018.

## Background

An incisional hernia is an abdominal hernia that occurs at the site of a previous surgical incision through which access to the abdominal cavity has been obtained and is the result of a failure of abdominal wall closure. The incidence of incisional hernia following laparotomy is historically quoted as up to 20%. It is estimated that in Europe over 80,000 incisional hernias become symptomatic annually but there is a lack of consensus regarding their optimal management [1]. Incisional hernias enlarge over time and can result in serious complications such as pain, bowel obstruction, strangulation and enterocutaneous fistula. Furthermore, the quality of life and chances of employment are reduced in patients suffering with an incisional hernia [2].

Recurrence rates in the management of incisional hernia remain unacceptably high; reports as high as 33% after first repair and 44% after second repair [3]. This has significant implications in terms of morbidity and socioeconomic cost. Recurrence rates are comparable for open and laparoscopic methods of repair [1].

It is now accepted that only relatively small (less than 3 cm) incisional hernias should be repaired via primary tissue approximation with sutures alone [4]. Open mesh techniques commonly used for larger hernias include the sublay (Rives-Stoppa) operation and the onlay (Chevrel) operation. The sublay technique has been reported to be more complicated and time consuming than the onlay repair but reports suggest a lower incidence of wound infection and seroma formation [2]. Wound complications after onlay repair may more rapidly lead to mesh infection because of the close and unprotected contact of the mesh with the subcutis. Uncontrolled studies have however also shown a low incidence of wound complications with the onlay method [4].

Laparoscopic repair is not always possible for large hernias. The size of the defect or the proximity of the defect to the costal margin or pelvis may render laparoscopic mesh placement difficult or unsuitable [1, 5]. Undertaking fascial closure prior to mesh placement is becoming more common place, with an emerging consensus that a hernia of up to 10 cm in diameter can be repaired using the laparoscopic technique [6]. These techniques include the use of intraperitoneal onlay mesh (IPOM) using a specially designed composite material secured with either spiral tacks or sutures. This type of mesh has been designed specifically to ensure good tissue integration whilst minimizing the risk of visceral attachments.

Goodney et al., identified 83 studies comparing open and laparoscopic incisional hernia repair from a structured Medline search [7]. This resulted in an overall comparison of 390 patients having open repair with 322 patients having laparoscopic repair. Perioperative complications and length of stay were reduced in the laparoscopic group. Frantzidees et al. identified 53 studies with a total of 5227 laparoscopic incisional hernia repairs; the rate of hernia recurrence was 3.98% [5]. This result was mostly the product of specialty centers in which minimally invasive surgery is prominent; the authors concluded that the true recurrence rate is likely to be higher. One of the first prospective randomised trials comparing open and laparoscopic repair of incisional hernia concluded that laparoscopic repair is associated with fewer, albeit more severe, complications and may improve some patient-centered outcomes [8].

Post-operative pain assessment has been examined as mesh-fixation with tacks is regarded as particularly painful. Reynvoet et al. demonstrated, upon examination of thirteen studies using either tack or suture fixation, that there was no real difference in pain score or recurrence [9]. However, no strong conclusions can be drawn from what is very heterogeneous data. With any mesh-fixation technique, transfascial sutures or the use of tacks, there remains a risk of chronic pain due to nerve damage as well as mesh migration. Although the tensile strength of sutures has been shown to be greater, in a laparoscopic pig model, no fixation technique was shown to be superior [10]. A randomised trial published in 2009 compared three fixation techniques; absorbable sutures with tacks, double crown technique with tacks only and non-absorbable sutures with tacks [11] (Table 1). The question here was whether sutures were better in combination with tacks or tacks alone, note that the fascial defect was not closed in any arm. No technique was found to be superior with the primary end-point being pain. Muysoms, in 2013, compared in a trial setting, spiral tacks using a double crown technique with transfasical sutures with a single crown technique, no fascial closure was attempted in this study with a leaning towards reduced recurrence with the double crown technique [12].

**Table 1:** A summary table depicting the randomised controlled trials undertaken to date comparing differing mesh fixation techniques in laparoscopic incisional hernia repair using intraperitoneal (IPOM) mesh

Author	Year	Number	Comparison	Result	Conclusion
Wassenaar et al. [11]	2010	199	3 groups: 1) Absorbable sutures with tacks, 2) Double crown with tacks, 3) Nonabsorbable sutures with tacks No FASCIAL CLOSURE	No difference with any group	More tacks with DC group and it was quicker (*p* = 0.03)
Muysoms et al. [12]	2013	76	Double crown technique DC v sutures and single crown technique SC	DC group operating time was shorter (*p* = 0.014), DC also had a reduced pain score at 4 weeks and at coughing (*p* = 0.028/*p* = 0.013), also at 3 months (*p* = 0.036)	DC technique was quicker and provided less pain up to 3 months but there was no difference in recurrence
Bansal et al. [13]	2012	110	Tack v suture fixation	Higher cost with tacks (*P* < 0.001). Suture fixation more cost effective than tacks Qol No difference	Early post op pain preferredwith suture, but no other differences were noted

No difference in recurrence was shown in any study. No study included data following fascial closure. Studies comparing various mesh fixation techniques

The laparoscopic repair has been criticised for producing cosmetically worse results than the open repair. This is because the hernia sac is not excised and the defect not closed.

The method and choice of repair of incisional hernia remains one of ongoing debate. Key considerations in planning surgery include the size of the defect and its proximity to the costal margin and pelvis. Areas of interest in the debate include known post-operative complications, post-operative pain and rates of hernia recurrence. Additional considerations for the patient include that of cosmesis and post-surgery quality of life.

### Rationale

To date no randomised controlled trial has compared the use of absorbable or non-absorbable tack fixation for laparoscopic incisional hernia repair after fascial closure. This prospective randomised controlled trial proposes to assess pain scores as a primary measure to determine whether absorbable tacks may provide a benefit in reducing the risk of immediate and long-term pain. Additional evaluation will see whether the risk of serious adverse events (SAE) is affected. This information is needed to provide evidence of the optimal method for surgeons and policy makers.

## Methods

### Study design

A prospective, single-centre, observer and patient blinded (double blinded), fixed design randomised trial.

### Study setting and sponsorship

Manchester University Hospitals NHS Foundation Trust, within the National Health Service (NHS) in the United Kingdom (UK) is the trial sponsor and the trial will take place in the Manchester Royal Infirmary.

### Objectives

#### Primary objectives

- To compare pain measured at rest and at activity with a visual analogue scale (VAS) at day 30 post-operation incurred from using absorbable tack fixation versus non-absorbable tack fixation with a composite mesh for the repair of midline abdominal incisional hernia in adult patients.

#### Secondary objectives

- To examine changes in pain using VAS at days 1 and 6, 30 days and one year post-operation at rest and activity.
- To detect the presence of intermittent hyperaesthesia, burning sensation and or jabbing pain in the abdominal wall.
- To compare whether the method of fixation for an IPOM repair has an effect on the following outcomes (complications will be classified according to the Clavien Dindo):

1. Seroma formation at 30 days post operation
2. Length of postoperative hospital stay
3. Operating time
4. Mesh fixation time
5. Wound infection (SSI)
6. Hernia recurrence at 1 year
7. Time to return to normal daily activity and work
8. Health related quality of life (QoL)
9. Other adverse events

### Study population

#### Inclusion criteria

All adults undergoing elective incisional hernia repair for midline abdominal incisional hernia, with a defect of 3-10 cm in diameter.

Midline abdominal incisional hernia defined as any hernia that arises from a previous surgical incision in the anterior abdominal wall musculature providing that there is a minimum distance of 3 cm from the border of the defect to the costal margin or pelvis.

#### Exclusion criteria

- Patients less than 18 years of age, or unable to give informed consent
- Patients over 80 years of age
- Clinically small incisional hernia < 3 cm maximum diameter
- Recurrent incisional hernia
- Emergency procedures (for irreducible, strangulated or obstructed hernia)
- Procedure involving purulent inflammation (e.g. abscess); preoperative perforation of gastrointestinal, biliary or genitourinary tract; penetrating trauma > 4 h old) or contaminated (Non-purulent inflammation; gross spillage from gastrointestinal tract; entry into biliary or genitourinary tract in the presence of infected bile or urine; major break in technique; penetrating trauma < 4 h old; chronic open wounds to be grafted or covered) surgery
- Failure to close the anterior rectus sheath intraoperatively
- Pregnancy
- Prisoners
- Patients with a Body Mass Index (BMI) > 40 kg/m^2^
- Patients participating in any other study, whose concurrent participation in the TACK study might place them at undue risk or might confound the study data in the opinion of the chief investigators

### Study process

Following potential participant identification and recruitment from incisional hernia referrals in participating surgeons’ out-patient clinics the potential participant will be sent information about the study. A Patient Information Sheet (PIS) will be given to the participant as soon as the decision has been made to list the patient for incisional hernia repair. In all cases, patients will be given sufficient time to consider the implications of their participation (minimum of one week). Written informed consent to participate in the study will be obtained preoperatively, at the same setting where (normal) consent for the intended surgical procedure is obtained > 24 h prior to surgery, by the operating surgeon. Intraoperatively, the size of the hernia defect will be photographed next to a metric ruler as per the Surgeon’s Instructions. Standardised post-operative care will be used in keeping with the Surgeon’s Instructions. Pain assessments will be conducted at pre-operation then post operatively on days 1, 5–7, day 30 (primary end point) and 1 year.

### Trial randomisation

There will be two arms to the trial (*n* = 136):

1. Mesh placement with absorbable tack fixation.
2. Mesh placement with non-absorbable tack fixation.

Participants will be allocated in sequence to a unique identification number and randomised to one of two groups i.e. receive either absorbable or non-absorbable tack fixation, stratified by defect size (3-6 cm or > 6-10 cm). The participant randomisation procedure is shown below.

Patients who have given consent and are due to be operated on will be randomised by a sealed envelope randomisation.

The operating surgeon will select from the sealed envelopes the tack to be used for the TACKoMESH Trial on the morning of the patient’s planned surgery to obtain the randomisation allocation to treatment. The patient’s initials and date of birth will be entered into the randomisation allocation; a study number and the treatment allocation assigned to each patient. The study number and treatment allocation will automatically appear on the Study Randomisation Form. Following the appropriate procedure the completed online Study Randomisation Form will be printed by the operating surgeon, placed in a provided sealed envelope, and labelled as ‘TACKoMESH - Study Randomisation Form and Incisional Hernia Repair Operation Note’.

### Surgical technique

All cases will be performed with a Consultant surgeon scrubbed throughout the entire case. Prophylactic antibiotics will be given at the time of the induction of anaesthesia. Pneumoperitoneum will be established according each surgeon’s preferred method. Three ports will be used (2 × 11 mm and 1 × 5 mm).The fascial defect will be measured in cm when the adhesions have been dissected free with no pneumoperitoneum present.

Adhesiolysis will be carried out with intrabdominal pressure at 12-15 mmHg. Fascial closure will be achieved using a No 1 Loop Maxon at no more than 10 mmHg pressure (8–10 mmHg).

The Mesh used should be fixated with the choice of fixation device that the patient has been randomised to without the use of transfascial sutures ensuring that at least 3 cm cover has been achieved beyond the defect. The size of the mesh used will be also recorded and fixation will be applied at the edges of the fascial defect with the remaining tacks placed on the outer & inner rim (along the closure line) to secure the mesh and prevent folding; the double crown technique. The number of tacks used will be recorded.

In case of intra-operative contamination, the laparoscopic procedure may be abandoned and converted to open surgery.

The use of local anaesthetic (1% lidocaine or 0.25%/ 0.5% bupivicaine) infiltration is permitted and must be recorded on the CRF form. Skin closure using subcuticular 3.0 monocryl or skin staples ± skin glue cover is permitted and must be recorded on the CRF form. Wound dressings may be applied according to the surgeon’s usual practice at the end of the procedure and must remain unchanged for 24 h unless there is a clinical indication for changing the dressing.

### Blinding

In order to ensure continued blinding of the research team, once the surgical procedures have been completed, the Study Randomisation Form will be placed in a sealed and labeled envelope, and securely inserted into the patients’ hospital notes. This may be opened in emergency if necessary to assist in the patient’s continuing management, or if the patient returns to theatre should a re-operation be required. Theatre staff will be instructed not to divulge the patient’s randomisation allocation to anyone.

### Sample size

Primary outcome: Pain score at rest and activity at day 30 recorded using visual analogue pain score (VAS) (0–10).

Previous studies indicate that a good approximation for the standard deviation (SD) in VAS is 2. With an alpha of 0.05, 80% power and 20% dropout, a sample size of 74 (37 per group) is sufficient to detect a difference of 1.5 between the groups.

There has been a suggestion that a rate of attrition of 20% is un-necessarily high for this group of patients, and we also consider whether the difference is achievable, therefore we recalculate the sample size considering these changes:

With a power of 80%, an alpha of 0.05, SD 2 and dropout of 5%, a sample size of 136 (68 per group) is sufficient to detect a difference of 1 between the groups.

No interim analysis will be undertaken therefore no adjustment is required in the sample size. This is a fixed design and no sample size re-estimation will be performed during the course of the study.

### Outcomes

#### Primary outcome

Pain score at rest and activity at day 30 recorded using visual analogue pain score (VAS) (0-10 cm).

#### Secondary outcomes

1. Visual analogue pain score (VAS) pain score at days 1, 5–7, 30 days and 1 year post-operative. A preoperative VAS score will also be recorded
2. Seroma formation (no fixed time point) (a seroma will be defined as any clinically apparent fluid collection at the site of mesh placement and classified as per the European Hernia Society classification system)
3. The length of postoperative hospital stay
4. The time to return to normal daily activity
5. Wound infection
6. Operating time
7. Mesh fixation time after adhesions have been taken down and fascia closed
8. Hernia recurrence at one year and at all time points
9. Health-related quality of life - assessed using the Short Form 36™ (pre-operatively, 30 days and 1 year), and the Carolinas Comfort Score™ (which is specifically designed for hernia repair) (at 30 days and 1 year).
10. Adverse events.

Wound and pain assessments at all time-points will utilise a standardised proforma. These wound assessment tools will be pre-validated on patients undergoing any abdominal surgery in the unit for reproducibility and ease of use prior to full trial launch.

### Statistical analysis

All analyses will be pre specified prior to data collection and conducted according to the intention-to-treat principle and presented in accord with CONSORT guidelines for the reporting of RCTs. All analyses will be conducted using R v3.0.2. All statistical tests will be 2-sided, and deemed to be statistically significant if *p* ≤ 0.05. The analyst will be blind to group allocation.

The primary outcome, VAS pain scores at day 30, will be explored via descriptive statistics and graphics. The scores in each of the groups will then be compared using an ANOVA with study group and defect size as factors. Secondary analyses will report all outcomes at each time-point using appropriate (parametric/non-parametric) descriptive statistics (frequencies [%] or mean/median [standard deviation (SD)/interquartile ranges (IQR)]) and graphics. The pre-operative variables will also be described via these methods.

VAS scores over time will be explored via a generalised linear model, using time, group and an interaction term as covariates. Operation time, mesh time, length of stay and time to return to normal daily activities will be compared between groups using descriptive statistics and independent t-test or Mann-Whitney U test as appropriate. Descriptive comparisons will also be made between the two defect size groups for each outcome. For secondary outcomes consideration for multiple testing will be included in the conclusions. Total numbers with any of seroma formation, wound infections or hernia recurrence at 30 days and 1 year will be reported with odds ratios.

## Discussion

Symbotex™ Composite Mesh is specifically designed to incorporate a monofilament polyester mesh with an absorbable collagen film. The collagen film acts to prevent bowel adhesions and subsequent complications, such as fistula formation, and thus provides a mesh safe for intraperitoneal use [10].

A current focus in the discussion on laparoscopic management of incisional hernia repair is the pain that patients experience after surgery. It is though that the tacks used to fixate the mesh are a contributing factor and many methods have been trialed to reduce the pain. TACKoMESH will attempt to determine whether the use of a more perpendicular fixation technique to the abdominal wall will help reduce the pain experienced by patients and hence enhance their recovery, which will inevitably reduce the amount of analgesia used in the post-operative period as well as the net benefit to the health of the economy.

All previous studies [11–13] have described the use of a non-articulating mesh fixation device. The Study uses a device with an articulating arm (ReliaTack™) and will therefore provide information on any possible variances in effect at operation; mesh fixation time and other intraoperative ergonomics. More importantly it will demonstrate whether a more perpendicular trajectory of tack to peritoneum will translate into less post-operative pain.

As no study to date has examined the effects of an articulating arm it is impossible to predict the outcome of the Study. Power calculations have taken many factors and permutations into consideration and a decision made that, to detect a difference of 1 between the two groups with a dropout rate of 5%, 136 patients will need to be randomized.

Secondary outcome measures were decided as per previous trials with the knowledge that seroma formation and classification [11–13] will be a necessary morbidity that requires documentation. This will be done as per a recently established classification system designed specifically for patients undergoing laparoscopic ventral hernia repair [14].

To the authors knowledge this is the first randomized trial for laparoscopic incisional hernia that will involve fascial closure and although fixation with absorbable and non-absorbable tacks have already been compared, this will have the novel addition of one of the fixation devices having an articulating arm.

Thus, the use of ReliaTack™, as a one-of-a-kind mesh fixation device with articulating arm that can deploy spiral absorbable tacks in the TACKoMESH study aims to provide level one evidence on the safety, efficacy and ease of use of this product as well as its effects on post-operative pain and other clinical observations and outcomes.

In conclusion, TACKoMESH is a randomised controlled trial that will provide important information on the effects of repairing an incisional hernia after fascial closure with an IPOM mesh (Symbotex™ Composite Mesh). It will compare the use of an articulating, mesh-fixation device that deploys absorbable tacks with a straight-arm, mesh-fixation device that deploys non-absorbable tacks measured by pain at 30 days and an overall assessment of the safety and efficacy.

## Abbreviations

ANOVA: Analysis of variance
BMI: Body mass index
CRF: Case report form
DC: Double crown
IPOM: Intraperitoneal On-lay mesh
IQR: Interquartile range
NHS: National Health Service
PIS: Patient information sheet
QoL: Quality of life
RCT: Randomised controlled trial
SAE: Serious adverse effects
SC: Single crown
SD: Standard deviation
SSI: Surgical site infection
UK: United Kingdom
VAS: Visual analogue score

## References

[1] Kingsnorth A, Banerjea A, Bhargava A (2009). Incisional hernia repair - laparoscopic or open surgery?. Ann R Coll Surg Engl.

[2] den Hartog D, Dur AHM, Tuinebreijer WE, Kreis RW. Open surgical procedures for incisional hernias. Cochrane Database of Systematic Reviews 2008, Issue 3. Art. No.: CD006438. 10.1002/14651858.CD006438.pub2. 10.1002/14651858.CD006438.pub2 PMC8924951 18646155

[3] Langer S, Christiansen J (1985). Long-term results after incisional hernia repair. Acta Chir Scand.

[4] Kingsnorth A (2006). The management of incisional hernia. Ann R Coll Surg Engl.

[5] Misiakos EP, Machairas A, Patapis P, Liakakos T. Laparoscopic Ventral Hernia Repair: Pros and Cons Compared With Open Hernia Repair. JSLS. 2008;12(2):117–25. PMC3016190 18435882

[6] Nguyen DH, Nguyen MT, Askenasy EP, Kao LS, Liang MK (2014). Primary fascial closure with laparoscopic ventral hernia repair: systematic review. World J Surg.

[7] Goodney PP, Birkmeyer CM, Birkmeyer JD (2002). Short-term outcomes of laparoscopic and open ventral hernia repair: a meta-analysis. Arch Surg.

[8] Itani KM, Hur K, Kim LT, Anthony T, Berger DH, Reda D (2010). Veterans affairs ventral incisional hernia investigators. Comparison of laparoscopic and open repair with mesh for the treatment of ventral incisional hernia: a randomized trial. Arch Surg.

[9] Reynvoet E, Deschepper E, Rogiers X, Troisi R, Berrevoet F (2014). Laparoscopic ventral hernia repair: is there an optimal mesh fixation technique? A systematic review. Langenbeck's Arch Surg.

[10] SymbotexTM clinging effect observed during the design validation conducted by Covidien in porcine model in May 2013- covidien internal memorandum 0901CR261a (July 2013) http://exhibitors.globalcastmd.com/files/download/b9c9c215572322b.

[11] Wassenaar E, Schoenmaeckers E, Raymakers J, van der Palen J, Rakic S (2010). Mesh-fixation method and pain and quality of life after laparoscopic ventral or incisional hernia repair: a randomized trial of three fixation techniques. Surg Endosc.

[12] Muysoms F, Vander Mijnsbrugge G, Pletinckx P, Boldo E, Jacobs I, Michiels M (2013). Randomized clinical trial of mesh fixation with “double crown” versus “sutures and tackers” inlaparoscopic ventral hernia repair. Hernia.

[13] Bansal VK, Misra MC, Babu D, Singhal P, Rao K, Sagar R (2012). Comparison of long-term outcome and quality of life after laparoscopic repair of incisional and ventral hernias with suture fixation with and without tacks: a prospective, randomized, controlled study. Surg Endosc.

[14] Morales-Conde S (2012). A new classification for seroma after laparoscopic ventral hernia repair. Hernia.

